# Barriers and facilitators to patient utilization of noncommunicable disease services in primary healthcare facilities in Nepal: a qualitative study

**DOI:** 10.1186/s12913-025-13050-8

**Published:** 2025-07-01

**Authors:** Sushmita Mali, Elizabeth C. Rhodes, Chandani Singh Nakarmi, Soniya Shrestha, Aarati Dhakal, Alina Bharati, Anupama Bishwokarma, Asmita Adhikari, Bikram Poudel, Binuka Kulung Rai, Sangita Manandhar, Surakshya KC, Dinesh Timalsena, Sashi Silwal, Meghnath Dhimal, Phanindra Prasad Baral, Felix Teufel, Sanju Bhattarai, Donna Spiegelman, Archana Shrestha

**Affiliations:** 1 Institute for Implementation Science and Health, Kathmandu, Nepal; 2 Hubert Department of Global Health, Rollins School of Public Health, Emory University, Atlanta, USA; 3 Emory Global Diabetes Research Center, Emory University, Atlanta, USA; 4 Center for Methods in Implementation and Prevention Science, Yale School of Public Health, New Haven, USA; 5 Department of Public Health, Kathmandu University School of Medical Sciences, Dhulikhel, Nepal; 6 Nepal Health Research Council, Kathmandu, Nepal; 7 Epidemiology and Disease Control Division (EDCD), Department of Health Services (DoHS), Ministry of Health and Population (MoHP), NCD and Mental Health Section, Government of Nepal, Kathmandu, Nepal; 8 Norwegian University of Science and Technology, Trondheim, Norway; 9 Department of Statistics and Data Science, Yale University, New Haven, USA; 10 Department of Chronic Disease Epidemiology, Yale School of Public Health, New Haven, USA; 11 Research and Development Division, Dhulikhel Hospital-Kathmandu University Hospital, Dhulikhel, Nepal

**Keywords:** Package of essential NonCommunicable diseases (PEN), Non-communicable diseases, Patient’s perspective, Health belief model (HBM), Perceived barriers, Perceived facilitators

## Abstract

**Background:**

The Nepalese government endorsed and implemented the Package of Essential Non-Communicable Disease Interventions (PEN) by the World Health Organization (WHO) to prevent and manage four major non-communicable diseases (NCDs): cardiovascular disease (CVD), diabetes, cancers, and chronic respiratory diseases. This study explored barriers and facilitators to patient utilization of NCD services at primary healthcare facilities in Nepal.

**Methods:**

We conducted a qualitative study with a 35 purposive sample of patients living with one or more NCDs (hypertension, diabetes, chronic obstructive pulmonary disease (COPD/ asthma) who sought healthcare at primary healthcare facilities in 14 randomly selected districts in seven provinces in Nepal that implemented PEN. Trained qualitative researchers conducted in-depth interviews in person in a private setting using a semi-structured interview guide developed based on the Health Belief Model in the local language. The interviews were audio-recorded, transcribed verbatim, coded inductively and deductively, and analyzed by a framework approach using Dedoose software.

**Results:**

From the perspectives of patients, key facilitators of service utilization encompassed free medicines, low-cost services, geographical and financial accessibility, less waiting time, positive interactions with health service providers, experiencing improvements in their health conditions, and support from family and peers. Barriers to utilizing services included inadequate health services (e.g., lack of medications and equipment), inaccessibility and affordability, inadequate health-related information from health service providers, low knowledge of NCD care, and lack of reminders or follow-ups.

**Conclusion:**

Enhancing NCD service utilization is potentially attainable through interventions that address patients’ knowledge, self-motivation, and misconceptions. Furthermore, strengthening the availability and accessibility of crucial services such as laboratory investigations, medications, equipment, and the patient-provider relationship is crucial for the sustainable implementation of PEN.

**Supplementary Information:**

The online version contains supplementary material available at 10.1186/s12913-025-13050-8.

## Background

Non-communicable diseases (NCDs) pose a significant and growing global health challenge, accounting for 74% of deaths worldwide [1] with a substantial 77% of NCD-related deaths occurring in low- and middle-income countries (LMICs) [2]. Cardiovascular diseases, diabetes, chronic respiratory diseases, and cancers are among the leading causes of morbidity and mortality, placing immense strain on health care systems [2] and economies [3] particularly in resource-constrained settings. Addressing NCDs requires a robust and sustainable healthcare approach, with primary healthcare (PHC) serving as a critical platform, particularly in LMICs, where it serves as the first point of contact for the majority of the population [2]. Therefore, the World Health Organization (WHO) initiated the Package of Noncommunicable Disease Interventions (PEN), a set of cost-effective, evidence-based interventions for NCD prevention and management within the primary healthcare system [3]. Through standard protocols, PEN interventions focus on early detection, risk assessment, affordable treatment, and lifestyle modification counseling and ensuring improved access to essential NCD care, particularly in resource-limited settings.

Many LMICs in the region have attempted to integrate NCD services into existing PHC frameworks, where they faced numerous challenges including inadequate financial and human resources, limited availability of essential medications, weak referral mechanisms, and insufficient health policy prioritization [4]. Conversely, successful models have demonstrated the potential of community engagement, and task-shifting strategies in enhancing NCD care at the primary level [5]. In South Asia, where a high NCD burden [6] coexists with challenges such as weak health infrastructure, workforce shortages, and financial constraints, PHC often struggles to provide comprehensive NCD care [7] and healthcare non-utilization [8].

Nepal, an LMIC in South Asia, faces a rising NCD burden, which accounts for an estimated 66% of total deaths [9] and reflects many of these regional challenges while also showcasing unique contextual barriers and opportunities in addressing NCDs through PHCs. In response, Nepal made efforts to integrate NCD services into PHC facilities, guided by the National Health Policy and Multi-sectoral Action Plan and WHO PEN. However, integration of NCD services within PHC settings remains uneven, with gaps in health system readiness including service delivery, human resources, a consistent supply of essential medicines, and patient adherence to treatment regimes [10, 11]. Furthermore, limited data exists on the effectiveness of interventions aimed at improving NCD service utilization among patients. While some factors influencing patient utilization have been identified, there remains a dearth of understanding regarding the complex interplay of socio-cultural, economic, and individual determinants affecting healthcare-seeking behaviors in the context of NCDs [12]. Yet, factors influencing patient utilization of services are underexplored.

Given this knowledge gap, we conducted a qualitative study exploring the factors influencing patient utilization of NCD services within primary care health settings from the perspectives of NCD patients, guided by the health belief model (HBM). The insights gleaned from our investigation will play a pivotal role in formulating demand-side strategies for meaningful advancement in LMIC healthcare systems for improved population health.

## Methods

### Theoretical model

**Fig. 1:**
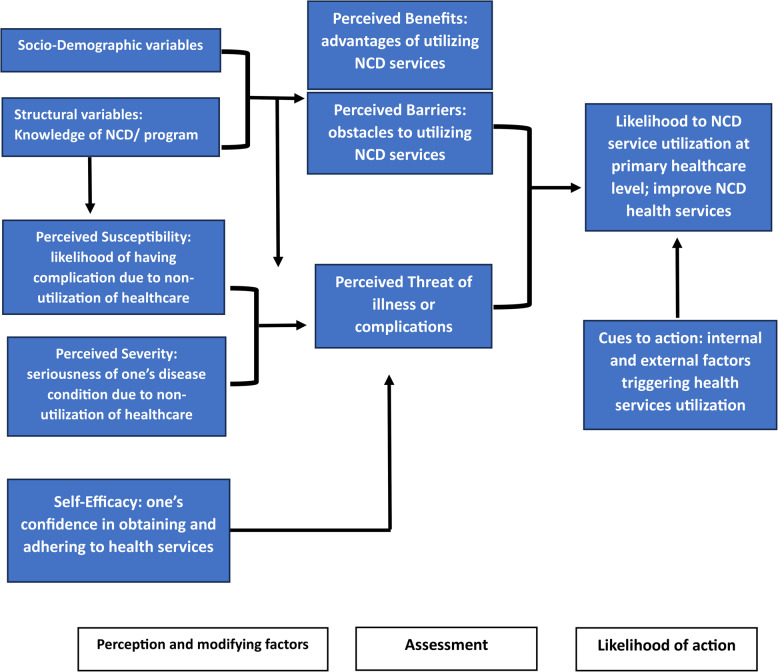
An illustration of the Health Belief Model influencing patient utilization

To guide this study, we employed the Health Belief Model (HBM), which explains NCD health service utilization through individual perceptions and modifying factors. Key constructs include perceived susceptibility, severity, benefits (facilitators), and barriers, as well as self-efficacy and cues to action, all of which influence the perceived threat and likelihood of utilizing NCD services [13, 14]. The design was well-suited to capturing these constructs at the individual level, with the primary healthcare setting providing the context for exploring service readiness and patient uptake through in-depth perspectives. The design focused on eliciting participants’ narratives related to socio-demographic influences, e.g., age, sex, education, family history of NCDs (modifying factors), knowledge and awareness of NCD/programs (structural variables), experiences of risk and disease severity (susceptibility/severity), access and affordability (barriers), self-motivation and adherence (self-efficacy), and cues to action such as prior illness or health service providers’ advice were explored to understand the alignment with model components to assess the likelihood of NCD service utilization (Fig. 1). Applying the HBM provided a theoretically grounded lens to understand how these factors influence health-seeking behavior, offering critical insights for improving the responsiveness and effectiveness of NCD interventions.

### Study design and setting

We conducted a qualitative study in Nepal, a low and middle-income country in South Asia [15]. Nepal’s PEN Implementation Plan (2016–2020) was developed to align with the Multisectoral Action Plan for the Prevention and Control of NCDs (2014–2020) [16]. The WHO PEN intervention was adopted by Nepal, with the Epidemiology and Disease Control Division (EDCD) under the Department of Health Services being the primary agency responsible for its implementation. To enhance accessibility for Universal Health Coverage (UHC), PEN was initially introduced as a pilot project in two districts (Illam and Kailali) in 2017. Subsequently, during 2018/19, PEN’s scope was extended to cover 31 districts out of the 77 in Nepal [17]. The PEN program consists of evidence-based, cost-effective interventions for the prevention and management of hypertension, diabetes, asthma, chronic obstructive pulmonary disease (COPD), and breast and cervical cancer [18]. The national PEN program aims to improve NCD management through early detection in primary healthcare facilities, i.e., health posts and PHCCs at the primary care level.

#### Study site and participants

A total of 35 participants were purposively selected from PEN-implementing districts, including 11 participants from 11 PHCCs and 24 participants from 20 Health Posts. The participants have NCDs, defined here as hypertension, diabetes mellitus (DM), chronic obstructive pulmonary disease (COPD), and asthma, and were visiting the outpatient department during data collection. The inclusion criteria comprised participants aged 30 years or older and under the prescription of NCD medicines. We excluded those with severe mental and physical disabilities. We ensured diversity in the sample by recruiting participants with varying characteristics, including gender, level of education and family history of disease.

This study received ethical approval from the Ethical Review Board (ERB) of the Nepal Health Research Council (Registration no: 327/2020P) and Kathmandu University School of Medical Sciences (Approval no: 110/20). Interviewers obtained verbal and written informed Consent from each participant, explaining the study’s purpose and the right to withdraw. Participant names, transcripts, and audio files were securely stored and accessible only to the research team to maintain confidentiality.

#### Sample size

The sample size was determined based on code saturation and meaning saturation principles [19] indicating the point at which no new codes or themes emerge from the data. We conducted 35 interviews to capture diverse and rich information until no additional insights or themes emerged. Subsequently, the achievement of saturation was determined through discussions with the research team, indicating that the analysis had reached saturation point.

#### Participant recruitment

With the approval of health service providers, trained research assistants (RA) approached participants waiting for follow-up clinical consultations in the health facilities using an everyday conversation– rapport-building approach. Once the participants agreed to engage in further conversation, we provided a comprehensive explanation of the study’s purpose, procedures, confidentiality measures, and potential risks and benefits to all potential participants. Those who provided written Consent were subsequently enrolled in the study.

### Data collection

We conducted face-to-face in-depth interviews (IDIs) with participants, guided by a semi-structured interview guide (supplementary file 1) with questions derived from the aforementioned Health Belief Model constructs. It aimed to gather information on facilitators, barriers, and individual-level perceptions regarding NCD service utilization offered by the PEN program. The guide was finalized based on insights from the pilot test and team discussions.

Seven trained qualitative researchers (SM, CS, BP, AD, AA, AB, SK, BKR, SK) and research officers from the Nepal Health Research Council conducted in-depth interviews between April and October 2021. All interviewers were native Nepali speakers trained in qualitative data collection. All interviews were in Nepali, except for one that was conducted in Doteli. Interviews were audio recorded and took place in private and quiet healthcare facility spaces. After each day of data collection, we held debriefing sessions to discuss emerging issues and topics and refine the interview guides. The interview duration ranged from 15 to 47 min, some shorter due to the patient’s limited knowledge, hesitation, or time constraints.

### Data management and analysis

We analyzed the data in six stages of framework analysis outlined by Ritchie & Spencer [20]: transcription, familiarization, coding, identifying a framework, charting into a matrix, and interpretation. Thematic framework analysis guided by HBM constructs was employed. Research assistants transcribed all interviews verbatim and de-identified them for anonymity. The first author (SM) reviewed transcripts alongside audio recordings for accuracy. Data were imported into Dedoose (v9.0.54). SM and CS independently read one-third of the transcripts to familiarize themselves with the data and collaboratively developed a codebook incorporating inductive and deductive codes. The codebook was iteratively refined with input from a qualitative expert (ER) to ensure alignment with HBM constructs. Initial coding of three transcripts established inter-coder reliability, reaching 89.6%, after which the remaining interviews were coded independently. Excerpts were categorized to identify recurring themes. A matrix was then created to chart summarized data and illustrative quotations by category. Coders continuously discussed emerging codes and patterns, generating detailed thematic descriptions.

## Results

The participants’ characteristics are provided in Table 1. Thirty-five NCD patients were interviewed, with 54% being female and ages ranging from 35 to 92 years. Among them, 63% had hypertension, and 17% had diabetes. About 49% reported a family history of NCDs. Approximately 50% of the participants had no formal education, 34% worked in agriculture, and 37% identified as homemakers.

**Table 1:** Characteristics of study participants (*n* = 35)

Characteristics	*n* (%)
Age category	
35 < 44	3 (8.5)
45 < 54	8 (22.8)
55 < 64	6 (17.1)
65 < 74	8 (22.8)
75 years or older	4 (11.4)
Sex	
Female	19 (54.3)
Male	16 (45.7)
Education level	
No formal education	16(45.7)
Basic Literacy	7(20)
Primary education	6(17.14)
Secondary education	4(11.4)
Higher education	2(5.7)
Family structure	
Nuclear family	10 (28.5)
Joint/extended family	25 (71.4)
Type of NCDs	
Hypertension	22 (62.9)
Diabetes Mellitus	6 (17.1)
Chronic Obstructive Pulmonary Diseases	2 (5.7)
Hypertension and Diabetes Mellitus	3 (8.6)
Hypertension and Chronic Obstructive Pulmonary Disease	2 (5.7)
Family History of NCDs	
Hypertension	10 (28.5)
Diabetes Mellitus	4 (11.4)
Chronic Obstructive Pulmonary Diseases/Asthma	3 (8.5)
None	15 (42.8)
Others (arthritis, gastritis)	2 (5.7)
Occupation	
Agriculture	12(34.28)
Homemaker	13(37.14)
Foreign Employment (Migrant worker)	2(5.71)
Retired	1(2.85)
Bank	1(2.85)
None	6(17.14)

**Fig. 2:**
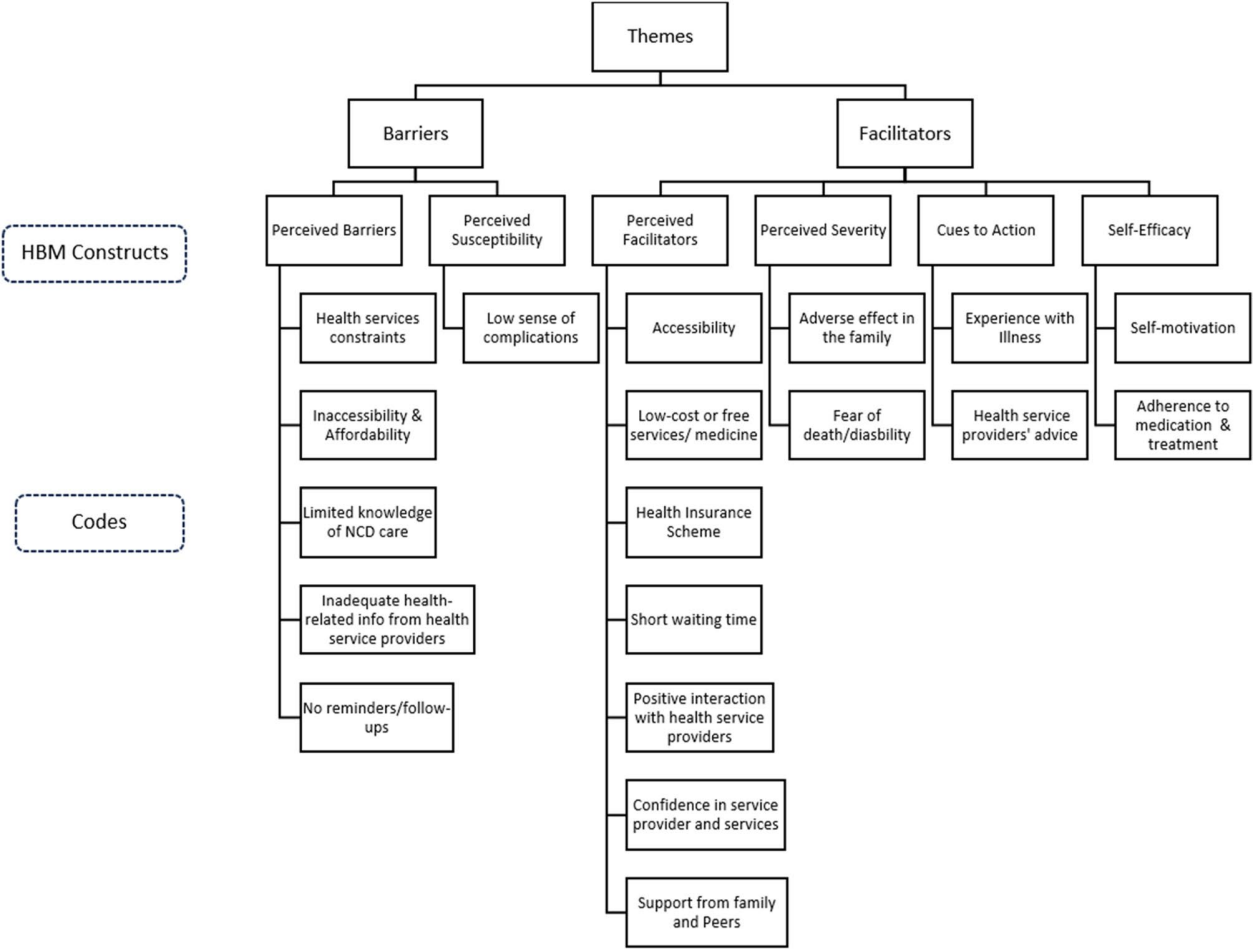
A diagram presenting themes and codes derived under the HBM constructs

The results are organized around the six constructs of the HBM: perceived susceptibility, perceived severity, perceived benefits, perceived barriers, self-efficacy, and cues to action (Fig. 2). The analysis reveals the factors shaping NCD service utilization, highlighting both the existing challenges and potential opportunities within primary healthcare settings.

### Perceived barriers

#### Health services constraints

All participants expressed frustration with the inadequate supply of medicines, lack of well-equipped infrastructure, and a shortage of skilled healthcare providers. The unavailability of essential drugs was a significant deterrent to NCD service utilization. Moreover, some participants encountered barriers in obtaining health insurance schemes, and those visiting health posts were often referred to higher-level centers for laboratory investigations and surgery, adding to the difficulties in accessing comprehensive care. NCD services are limited to blood pressure screening, blood glucose testing, and medication at health posts for individuals without health insurance.

*“The doctor is not available. To date, we have not received anything easily… we have to buy most of the medicines as they are unavailable in the facility… In case of serious health issues, they refer to bigger health facilities, hospital.”* (DM Patient, 35 years, Male, PHCCs)

*“Lab services like blood sugar tests for diabetes are not available in this health post*,* so we have to go to Beni Hospital. Medicines are not available; we have to travel a long distance for it.”* (DM Patient, 63 years, Male, HP)

Participants expressed frustration regarding the lack of access to necessary medications despite having paid for insurance. The participants highlighted encountering excuses from healthcare providers regarding medication availability or delays due to administrative procedures, such as meetings for decision-making, causing delays. This experience highlights systemic challenges within the healthcare system that hinder individuals’ ability to access essential treatments in a timely manner. Such barriers not only exacerbated the burden of chronic diseases but also contributed to feelings of disillusionment and dissatisfaction among patients who rely on insurance coverage for healthcare support.

*“Because we are suffering from chronic disease*,* that is why we have paid for insurance*,* but they do not provide medicines. When we ask for the medicine*,* they make excuses of unavailability or tell us they have to sit for a meeting and decide and stuff like that.”* (DM Patient, 50 years, Female, HP)

This was especially true for older participants who had difficulty managing chronic health conditions or in some cases, multiple chronic health conditions simultaneously, navigating complex healthcare services and coping with physical limitations hindering mobility and independence.

*“We have been getting our medicines through an insurance scheme from Gandaki Hospital*,* but we are old and cannot go there. Therefore*,* I have come here to ask if getting the medicines is possible*,* but it is not available here.”* (HTN Patient, 92 years, Male, PHCCs)

#### Inaccessibility and affordability

Geographical accessibility posed a significant obstacle to seeking NCD care, encompassing physical distance and limited transportation options. Participants residing in rural hilly areas expressed that seeking healthcare elsewhere was impractical. Moreover, the region’s natural hindrances, including intense seasonal rains and landslides, further impeded participants from accessing necessary services. Individuals attending health posts reported being directed to higher-level facilities for medication and laboratory work, resulting in additional travel expenses that imposed an extra financial strain. The cost associated with treatment played a pivotal role in shaping individuals’ health-seeking patterns. This often led to the difficult decision of either postponing, preceding, or discontinuing treatment, a sentiment particularly pronounced among participants from economically disadvantaged households.

*“During the monsoon season, due to heavy rain, the roads are blocked. It is difficult to travel during the season. If there were a graveled road, it would have been easy. Transportation problems and unavailability of vehicles is a big problem in our area.”* (HTN & COPD Patient, 67 years, Male, HP)

“*It takes hours to reach Beni Hospital… an hour for locals and 2 hours or more for those who are not from around. It takes a whole day to reach Beni*,* and it costs Rs. 200. That is a lot for us: bus fare*,* medicine costs*,* lunch expenses; the trip is very costly for us.”* (DM patient, 63 years, Male, HP)

Many patients struggled to adopt lifestyle changes, particularly due to challenges in modifying household cooking practices for a single individual, as family members were unwilling to compromise on taste. One older patient reported limited alcohol consumption at night, believing that small amounts would not be harmful.

“*The advice during counseling sessions is not feasible to follow*,* and I am not following it… Modification practices are not possible when you are the only person suffering from NCD*,* like food restrictions.*” (Pt4, 64 years, Male, DM, HP)

#### Limited knowledge about NCD care

The lack of awareness surrounding NCD programs, such as PEN and health insurance schemes, acted as a barrier, hindering participants from using available NCD services. Many participants were unaware that they required regular health checkups for NCD care and lacked comprehensive education on their health conditions and the corresponding management protocols. Certain participants held reservations about relying on pharmaceutical interventions indefinitely, leading them to start medication only after exploring all conceivable alternatives, including traditional and herbal remedies. A small minority expressed concerns about potential medication side effects, which subsequently discouraged them from maintaining consistent adherence to the prescribed treatment.

A prevalent lack of understanding regarding possible disease complications, combined with limited awareness of the inherent health risks and significant consequences, contributed to the delay in seeking necessary medical attention. Within this context, a subset of participants even reported instances of prematurely discontinuing medication once their symptoms improved.

*“I did not know about the drug information. If I had known that medicine for high blood pressure should be taken lifelong, I think I would not have started it.”* (HTN & DM Patient, 63 years, Male, HP)

*“I am unaware of the NCD services offered through the Health Facility by the Nepal Government. I heard about the PEN program today only…My hands and legs went weak. First*,* I got treatment with a witch doctor. That did not work. I became ill; I went to Kathmandu and came to know it was high blood pressure.”* (HTN Patient, 76 years, Male, HP)

#### Inadequate health-related information from health service providers

Communication breakdowns between participants and health service providers, coupled with shortcomings in delivering effective counseling, emerged as additional obstacles to the utilization of NCD services. Instances of subpar encounters where participants received insufficient information about their health conditions, exacerbated by limited doctor availability, particularly in PHCCs, were highlighted as demotivating factors. Some participants expressed dissatisfaction with the lack of counseling from health service providers regarding disease management, encompassing medication adherence and lifestyle adjustments. Several participants also indicated that their health service providers failed to provide insights into the various NCD-related services available within the healthcare facility.

*“While sharing the symptoms and difficulties, nobody gave information on the actual cause of high BP or the cause of breathlessness. I do not know what caused me HTN and Asthma… there has not been much counseling on my health conditions.”* (HTN & COPD Patient, 67 years, Male, HP)

“*I have not received any suggestions from Health service providers for my conditions. Doctors do not give much time. They only ask what is the reason for our visit and write prescriptions. They do not talk much.*” (HTN & DM Patient, 53 years, Female, HP)

#### No reminders/follow-ups

No follow-ups or reminders from health service providers emerged as an additional hindrance to accessing NCD services. Some individuals expressed that timely reminders from Health service providers could have averted these lapses in their follow-up routine. Participants highlighted instances of missing their scheduled follow-up visits due to conflicting priorities.

*“There has not been any contact from the health service providers…there is no follow-up service. When the medicine finishes and we do not feel well*,* we come in for checkups ourselves. Nothing is done from the health centers.”* (DM Patient, 63 years, Male, HP)

#### Perceived susceptibility

Few participants expressed a low sense of vulnerability and perceived themselves as unlikely to be affected, downplaying their risk despite exposure to risk factors in the absence of symptoms. A few participants demonstrated high susceptibility, recognizing the disease’s impact on anyone, its link to tobacco use, and their own past risky behaviors. With an improvement in their health condition, participants demonstrated a high perceived susceptibility, as evidenced by their motivation to utilize NCD care.

*“I do not feel any danger as I have not experienced anything bad till now by not going for healthcare checkups regularly.”* (DM Patient, 63 years, HP)

“*This disease can occur to anyone. I have seen many people suffering from it. Tobacco and smoking cause this disease. I used to smoke and take tobacco.”* (COPD Patient, 76 years, HP)

### Perceived facilitators

#### Accessibility

Most participants reported that the proximity of the health facility to their homes encouraged them to use available services. Participants living nearby or with easy access to health facilities reported seeking services regularly, as geographical accessibility played a significant role in NCD service utilization and continuity of use. The proximity of the health facility also reduced the need for follow-up calls, as they could easily access services when needed. A male diabetes patient mentioned that the proximity of the health post to his home allows him to visit easily during his free time, emphasizing the benefit of the 24-hour service availability, which facilitates access during emergencies. Similarly, a 92-year-old male hypertension patient appreciated PHCCs, highlighting their accessibility and convenience.

#### Low-cost or free services and medicines

The satisfaction of participants with NCD services depended on several factors, including free health checkups, facilitation of medicine refills, low medication costs, and low user fees for laboratory and diagnostic services. Many participants reported experiencing a positive outcome in their health condition due to the medication and diagnostic services they received from primary healthcare facilities.

*“I come here because medicine is available… it has been one year that I have been taking medicine from here, previously I used to buy medicines from outside.”* (HTN & DM Patient, 52 years, Female, HP)

*“The medicine from the health facility has helped to control and manage my condition. I have not visited other health facilities*,* taking medicines from here only.”* (DM & HTN Patient, age missing, Male, HP)

#### Health insurance scheme

The availability of free medicine and free health checkups or subsidized prices through social health insurance encouraged participants to seek treatment from health facilities, particularly those from economically disadvantaged backgrounds. The financial burden associated with healthcare services served as a significant barrier to accessing necessary treatment. The provision of free medications and screenings, along with subsidized prices through social health insurance, alleviated this burden, making healthcare more accessible and affordable. Participants reported that the health insurance scheme was only accessible at PHCCs and covered free consultations, limited medicines, and laboratory tests.

*“Insurance has opened access to all kinds of (health) checkups*,* medicines*,* and other health-related services. The treatment cost is cheap; I tested my sugar for Rs.40 ($0.03)… My sugar was diagnosed at this facility when I came to check uric acid.”* (DM Patient, 61 years, Male, PHCCs)

#### Short waiting time

A key factor positively influencing NCD service utilization was the practical waiting time of under 30 min per visit, which participants highly valued in their healthcare experiences. Several participants from both PHCCs and health posts expressed appreciation for the relatively short waiting times for services, including consultations, health examinations, and medicine dispensing. This efficient service delivery motivated them to seek healthcare services at the health facility.

*“I get the services on time*,* I do not have to wait*,* and they respond immediately… there has not been a time when I had to return without getting checked; someone is always available.”* (HTN Patient, 92 years, Male, PHCCs)

#### Positive interaction with health service providers

The willingness of service providers to listen to participants’ concerns and exhibit positive behaviors played a significant role in making participants feel comfortable and building trust. The existence of a strong patient-provider relationship emerged as a key motivating factor for participants to utilize services, including regular visits to healthcare facilities and undergoing screenings. Participants highly valued the information and guidance provided by healthcare providers on healthy diets and medications, enabling them to gain knowledge about disease management and a better understanding of their health conditions. They also emphasized the healthcare providers’ politeness and support.

*“They [health service providers] are accommodating, and respond nicely. They behave politely. The doctor is excellent here.”* (HTN Patient, 40 years, Female, HP)

“*Health Service Providers behave well*,* provide suggestions on the do’s and do not*,* being one of the reasons to visit the health facility.”* (DM Patient, Missing age, Male, HP)

#### Confidence in health service providers and services

Participants expressed trust in the healthcare facility and confidence in the services provided, citing reassurance from access to medications and the convenience of a local, government-run healthcare facility within their community.

*“I am confident in the services: first, it is near to my place; second, the health service providers attend to us very well and ask about our concerns… and then check our pressure. Instructions from him are doable and follow.”* (HTN patient, 40 years, Female, HP)

*“I feel that medicines and health services from here will help me to improve my health condition and live a healthy life.”* (HTN Patient, 50 years, Female, HP)

#### Support from family and peers

Family and peer support played a significant role in encouraging the utilization of healthcare services. Participants described that the moral support they received from family members, friends, and neighbors was crucial in motivating them to initiate treatment and start medication. The various forms of support provided by family and peers included encouragement for checkups, medication reminders, assistance with travel, especially for those with mobility challenges or transportation limitations, medication refills, financial support, and help with household chores such as cooking and cleaning. This encouragement and assistance contributed to better treatment adherence, increased health awareness, and improved health outcomes.

*“My husband took me to the Nepalgunj Hospital for treatment and got me medicine. He always takes me to the health facilities for checkups.”* (HTN Patient, age missing, Female, PHCCs)

*“At the time of sickness*,* without my friends*,* I might have lost my life when my BP (blood pressure) was increased. I recall a time when my friends looked after my cattle*,* letting me take a rest and seek health care.”* (HTN Patient, 61 years, Female, HP)

#### Perceived severity

Individuals’ perceptions of the severity of health conditions significantly influence their motivation to seek treatment, thus contributing to NCD services utilization. A few participants shared that witnessing their family members’ experiences of complications due to not regularly using medication or healthcare made them realize the severity of NCDs. Furthermore, seeing others suffer as a result of NCD complications like vital organ failure, disability, paralysis, or early death and associated morbidity and mortality due to delay in seeking healthcare made them fearful of NCDs. One participant who experienced two family members with NCD-related complications shared.

*“My brother had hypertension and did not see a doctor or use medication regularly… he was paralyzed and later died of complications. I remember what he had to go through…. I’m scared that I might die like him*,* too.”* (HTN Patient, 66 years, Female, HP)

Most participants reported that their experiences of symptoms, such as dizziness, heartache, headache, breathlessness, fatigue, difficulty breathing and walking, loss of appetite, and tingly sensations, influenced their decisions to seek medical help.

*“I used to have dizziness*,* pain in the neck*,* loss of appetite*,* body aches*,* and headache*,* so I had gone for checkups experiencing all these symptoms.”* (HTN Patient, 46 years, Female, HP)

### Cues to action

#### Experiences with illness

Observing family members endure severe conditions motivated participants to seek medical care proactively. Some participants noted that their commitment to medication adherence was influenced by the presence of family members with NCDs, as they had cared for them or witnessed the impact of NCD-related fatalities.

*“I have taken care of my grandmother for the past 12 years. I had to carry her inside and outside of the room/house. Once, she fainted, and she could not move half of her body. Seeking that, I am motivated to keep up with my clinical visits and medication.”* (HTN Patient, 66 years Female, HP)

“*My husband fell very ill; I had to take care of him*,* help him go to the toilet and clean up*,* and feed him. I went for a checkup because I could not sleep well at night. This all led me to start medicine.”* (HTN Patient, 60 years, Female, HP)

Participants added that their personal experiences and fear of complications or death heightened their awareness and commitment to managing their NCD conditions. Many participants stated that their fear of disease consequences prompted them to prioritize control measures such as regular monitoring, treatment, follow-ups, medical adherence, and lifestyle modifications, leading to greater use of NCD services.

*“I take medicine with the fear of losing my life. The doctor informed me that arriving just one minute late could have cost me my life. I worry about not getting medicines from here; I wonder who will provide them. As a result*,* I am now almost disabled and unable to be economically active.”* (HTN Patient, 50 years, Female, HP)

#### Health service provider’s advice

Most participants reported receiving guidance from their health service providers regarding medication adherence, regular checkups, adopting a healthy diet, and abstaining from alcohol and tobacco. Some participants indicated they were willing to pay for treatment when advised by their service providers. Experiencing health improvements as a result of following these recommendations positively impacted service utilization. A significant number of participants expressed a preference for revisiting health facilities when provided with dietary and lifestyle modification advice.

*“The health service providers advise avoiding oily, spicy foods and fatty meats. They helped me to be updated about my health. If my blood pressure reading is high, they suggest the dos and don’ts and encourage me to take medicine.”* (HTN Patient, 66 years, Female, HP)

“*The medicines from the doctor have improved my health and also*,* counseling and advice on diets like healthy foods*,* adding white meat instead of red meats*,* eggs*,* and more fluids*,* and avoiding oily*,* salty foods. I like coming back and consulting.*” (HTN Patient, 75 years, Female, PHCCs)

### Self-efficacy

#### Self-motivation

One reason that participants were motivated to adhere to treatment was their love for their family and their strong desire to live longer to witness the growth of their children’s offspring. To manage their health conditions, participants were also proactive and resourceful in the face of limited healthcare services.

*“They do not have a lab service to assess blood sugar levels. Therefore*,* I have bought the device. Sometimes my daughter-in-law or I do the checking in every 15 days.”* (DM Patient, age missing, Male, PHCCs)

#### Adherence to medication and treatment

Many participants stated that they promptly started taking medication after their diagnosis and experienced positive health benefits from it.

*“My pressure is better now. I feel that medicines and health services help me improve my health*,* and I am confident that I will live a healthy life.”* (HTN Patient, 85 years, Male, HP)

## Discussion

This qualitative study explores the barriers and facilitators to NCD service utilization at the primary healthcare level in Nepal, using the HBM as a guiding framework. While initiatives like the WHO-PEN package have contributed to strengthening NCD care at the primary level, several systemic and patient-related barriers continue to hinder effective service utilization. By examining six core HBM constructs: perceived susceptibility, perceived severity, perceived facilitators, perceived barriers, self-efficacy, and cues to action, the study analyzes participants’ perspectives to uncover the complex interplay of factors influencing healthcare-seeking behaviors. The findings highlight both opportunities for improvement and the ongoing challenges within Nepal’s primary healthcare system in addressing NCDs.

One of the key barriers identified is the health services constraints, including the limited availability of essential NCD services and medications at the health facilities, a challenge echoed in other LMICs [7]. Despite policy efforts, stockouts of essential medications, diagnostic instruments, and capacitated healthcare infrastructure [11] were common, leading to disruptions in care continuity. This is further exacerbated by the shortage of skilled healthcare providers, particularly in rural settings [21]. The NCD services provided were limited to basic screenings and medication in some instances, specific centers lacked screening programs necessary for diagnosing NCDs, a similar finding reported by a study in Nepal [22].

Other barriers to utilizing these services were insufficient information provided by healthcare providers, minimal to no counseling, the absence of reminders for medication and follow-up visits, and patient dissatisfaction with the services rendered. Similarly, in a study [23] participants indicated that the attitude of service providers and inadequate communication during consultations are some of the constraints in accessing healthcare services in rural South India [24] and they perceived health workers as being responsible for and most knowledgeable about the patient’s health.

From the participant’s perspective, inaccessibility (geographical) and affordability emerged as significant deterrents to service utilization, a finding also reported in another study in Nepal [10]. Out-of-pocket expenses for medications, lab tests, follow-up visits, and transportation impose hardship, particularly on economically poor populations. These findings align with studies from South Asia that highlight financial barriers as a significant determinant of healthcare access [10, 25, 26]. The scenario reflects a broader trend observed in LMIC [27] where health disparities and out-of-pocket health expenditures are rising, and Nepal is no exception [28, 29]. The financial burden tends to escalate with the number of coexisting health conditions, contributing to increased out-of-pocket expenditure with the number of co-morbidities [30]. Similar observations were noted in a study conducted in India, where the cost of medication constituted the most substantial proportion of expenses for participants, followed by spending on healthcare providers [31].

Cultural Beliefs and perceptions regarding NCDs also played a crucial role in shaping health-seeking behaviours. Participants who refrained from seeking healthcare often did not perceive hypertension as a serious medical condition [32]. Similar patterns have been observed where the asymptomatic nature of the disease contributed to a delay in initiating healthcare-seeking behavior, with individuals prioritizing symptomatic treatment approaches [33, 34]. In our study, participants also had inadequate knowledge and misconceptions about NCD treatment, which made them hesitant to make a lifetime commitment to medication. Similar findings have been observed in studies from Nepal [35, 36] and LMICs [24, 37, 38]. Individuals lacking literacy skills often encounter challenges in accessing relevant information and knowledge [39] impeding the timely initiation of disease treatment and control [40]. Furthermore, people with less education tend to favor alternative treatment modalities [35, 37] a phenomenon also prevalent in other LMICs [41].

Despite the challenges, our study also identified several facilitators that enhanced patient service utilization. Receiving general health services and availability of free medicines encouraged participants to visit primary healthcare facilities, which led to overall satisfaction with the service provided [42]. The presence of a social health insurance scheme that comprehensively covered healthcare costs and the availability of medications served as motivating factors for individuals seeking NCD care at health facilities. Similarly, the minimal waiting time for consultations, health examinations, and medication dispensing enhanced service satisfaction, encouraging participants to visit healthcare facilities regularly. The findings align with a study in Iraq [43]. Furthermore, the behavior and conduct of healthcare providers significantly influence patient perceptions of health services. Similar findings were observed that establishing rapport and fostering trust with participants positively impacted their perception of the quality of care [44].

Proximity to healthcare facilities emerged as a critical factor in facilitating service utilization at the primary healthcare level in Nepal. Geographical barriers limited service utilization; however, in hilly areas, nearby health facilities were crucial in reducing travel time, costs, and logistical challenges, thereby ensuring easier access to care. This finding is unsurprising and aligns with what is often observed in the healthcare system worldwide [45, 46]. Similar patterns were observed in studies conducted in various other countries such as India [24] and Pakistan [47].

Fear of complications or death, experiences of illness within the family, past negative experiences or deaths, and the presence of other conditions (co-morbidity) were factors encouraging individuals to seek healthcare and treatment adherence, underscoring the severity of the diseases. These findings align with similar studies conducted in India [48] and Nigeria [49] suggesting that perceived severity acts as a motivator for medication adherence. Furthermore, participants who received support from their families and peers played a vital role in NCD healthcare utilization and enhanced medication adherence. This aligns with findings from previous studies in India [44] and other South Asian countries [38] where medication adherence, regular medicine refills, and hospital visits were positively associated with receiving family and peer support. Additionally, certain cues to actions such as medicine monitoring by family members, healthcare provider’s advice, and self-efficacy motivate participants to prioritize treatment adherence. Like our study findings, studies have also emphasized the role of family member support in the utilization of hypertension medical care and highlighted the importance of educating families and communities [50, 51].

Existing research on NCD service utilization in Nepal has primarily centered on the viewpoint of health service providers [10, 52] and health authorities [53]. The findings from our study’s patient-specific challenges highlighted unique contextual factors that require targeted interventions. Similar studies have explored the perspectives of NCD patients in Nepal; however, constraints include the perspectives from patients and non-patients [54, 55]. Patient utilization matters profoundly as it directly impacts the timely detection, management, and prevention of NCDs, thus influencing health outcomes and overall healthcare system efficiency. A comprehensive understanding of how patients perceive and engage with NCD services is crucial to refine their NCD care models. By addressing the unmet needs of patients and fostering greater patient involvement in their care, these models can contribute to improved outcomes and a higher quality of care [12, 56].

### Strengths

A key strength of this study is its focus on the understudied patient perspective on NCD service utilization in Nepal. Using an iterative analysis approach with code saturation, it captured diverse perceptions and emerging issues. The inclusion of participants from all seven provinces further strengthens the geographic representativeness and relevance of the findings, offering a comprehensive understanding of contextual facilitators and barriers to NCD service uptake across diverse settings.

### Limitations

This study has a few limitations. First, the findings may not be generalizable to individuals who sought care outside the selected primary healthcare facilities, as recruitment was limited to these settings. Second, participants included those with diabetes, hypertension, and COPD but not cancer patients. Third, the HBM, while useful, focuses primarily on individual perceptions and may not fully capture the broader socioeconomic and environmental factors that influence healthcare access and utilization. Lastly, the interviews were conducted at the health facilities, so participants may have been reluctant to report some of the health-worker related barriers due to social desirability bias.

## Conclusions

In conclusion, improving NCD service delivery and patient utilization at PHC facilities in Nepal requires a multifaceted approach that addresses systemic, financial and socio-cultural barriers. Addressing the challenges of significant gaps in service delivery and patient utilization requires a multi-sectoral approach involving policy reforms, capacity building, and community-driven initiatives to ensure equitable and sustainable NCD care at the primary level. Policy efforts should focus on strengthening the availability of essential NCD medications and treatments, as well as investing in human resources and infrastructure. Simultaneously, comprehensive health education is needed to raise awareness about the availability of healthcare services, establish community outreach for remote areas, promote patient empowerment, and leverage innovative care delivery models through multi-sectoral collaboration. Moreover, further comparative research across diverse LMIC settings is needed to tailor interventions that address both systemic and culturally specific challenges.

## Supplementary Information

Supplementary Material 1.

## Data Availability

The datasets used and/or analysed during the current study are available from the corresponding author on reasonable request.

## Abbreviations

CVD: Cardiovascular Diseases
COPD: Chronic Obstructive Pulmonary Disease
DM: Diabetes Mellitus
DOHS: Department of Health Services
EDCD: Epidemiology and Disease Control Division
HP: Health Post
HA: Health Assistant
HTN: Hypertension
HSP: Health service providers
HBM: Health Belief Model
IDI: In-depth Interview
MoHP: Ministry of Health and Population
NCD: Non-communicable Diseases
NHRC: Nepal Health Research Council
PEN: Package of Essential noncommunicable diseases
PHCCs: Primary Health Care Centers
RA: Research Assistant
UHC: Universal Health Coverage
WHO: World Health Organization
