# Supplementary material for: Barriers and facilitators to patient utilization of noncommunicable disease services in primary healthcare facilities in Nepal: a qualitative study
Source: BMC Health Serv Res. 2025 Jul 1;25:863. doi: 10.1186/s12913-025-13050-8 (PMC12217986; doi:10.1186/s12913-025-13050-8)
Supplement: Supplementary file 1 — Supplementary Material 1. [file 12913_2025_13050_MOESM1_ESM.docx]

**Additional file**

**Interview guide for NCD Patient**

Introduction

Namaste!

My Name is ……………, and we are conducting a study to assess implementation of PEN program in Nepal, and to explore the factors that support and hinder its implementation. Thank you for agreeing and taking the time to speak with us today.

Through this conversation, we hope to understand your experiences and perspectives. Your thoughts are valuable to us, and we encourage you to share openly.

We will be recording the interview for analysis purpose and also take notes. If at any point you prefer not to be recorded, please let us know. Everything you share will kept strictly confidential and used only by our research team for the purposes of this study.

The interview will take approximately one hour, beginning with some initial questions.

**Interviewer Instructions:**

1. Ensure informed consent is obtained.
2. Verify that the recording device is on.

Everything you say is now being recorded.

|  | **Questions** | **Probes** |
| --- | --- | --- |
| **Warm up- Introductory Questions (3 minutes)** | 1.Please tell us about yourself? | -Age, Marital status, Education, Occupation, family structure and members, disease type, family history of any NCDs |
| **Awareness of disease and PEN services/Program (10 minutes)** | 2.Would you tell us something about your disease? | -Non-communicable diseases/ Communicable diseases, cause, signs & symptoms |
|  | 3.How was your disease diagnosed? Please explain briefly about it. | -How did you feel when you first noticed the symptoms of your disease?  -What kind of suggestions did the health service provider give you? |
|  | 4.What are your reasons for seeking care in this health facility? | -How did you end up in this health facility?  -Where do you usually go for treatment?  -Why do you prefer to go (health facility)? |
| **Perceived susceptibility (6 minutes)** | 4. What do you think could increase the chances of a person to develop  ……….. [DISEASE]? |  |
| **Perceived severity (6 minutes)** | 5. What health problems can your disease lead to ? | -Any challenges or impacts of it has brought in your life? |
| **Perceived benefits (6 minutes)** | 6. What do you think are the benefits of PEN/NCD services provided by this health facility? | -In your view, as a patient, how important is it to have program for NCD services/PEN program?  -Can you share any positive changes it has brought in your life? |
| **Perceived barriers (6 minutes)** | 7. How accessible are the health/NCD services provided by this health facility for you? | -How accessible are the medications?  -How accessible are the health service providers during the time of emergencies?  -What is the greatest challenge in accessing the services? |
|  | 8.What makes it difficult for you in accessing NCD services in this health facility? | -Why? What makes it hard to access services? |
| **Perceived facilitators (6 minutes)** | 9. What helps you in accessing NCD services in this health facility? | -Why? What makes it easy to access services? |
|  | 10. How satisfied are you with the services provided by this health facility? | -Why? How helpful has it been in improving your health? |
|  | 11. How does your health service provider help you for follow up visit? | -What happens when you miss a scheduled visit?  -How does your health worker contact you when you miss a scheduled visit?  -How does it help to manage your illness when they contact you? |
|  | 12. How do you think we can improve services for NCD? |  |
| **Cues to Action (6 minutes)** | 13. What prompts you to seek care for your disease condition from this health facility? | -Whom do you reach out for help?  -How supportive are they while you are seeking care for your disease?  -What is your family support like while you are receiving care?  -What is your peer support like while you are receiving care? |
| **Self-efficacy (6 minutes)** | 1. How confident are you that you can receive care for your disease from this health facility? 2. How confident are you that you can follow the instructions for managing [DISEASE] provided by the health service provider? | -How helpful were the counseling services provided by your health service provider for the management of your disease? |
| **Wrap Up Questions (5 minutes)** | 1. Overall, how useful have been the services in managing your disease? 2. What can be done to improve the NCD services and care in Nepal?   Now, we are at the end of our conversation. Do you have anything else you would to add to our discussion today? |  |

**Thank you for your time.**

**STOP RECORDING**
